# Long-Term Immunogenicity and Protection of a rHVT-H9/Y280 Vaccine Against H9N2 Avian Influenza Virus in Commercial Layers with High Maternal Antibodies

**DOI:** 10.3390/ani16020242

**Published:** 2026-01-13

**Authors:** Sang-Won Kim, Jong-Yeol Park, Ji-Eun Son, Kai-Qiong Zheng, Cheng-Dong Yu, Ki-Woong Kim, Won-Bin Jeon, Yu-Ri Choi, Hyung-Kwan Jang, Bai Wei, Min Kang

**Affiliations:** 1 Department of Avian Diseases, College of Veterinary Medicine and Center for Avian Disease, Jeonbuk National University, Iksan 54596, Republic of Korea; 2 Bio Disease Control (BIOD) Co., Ltd., Iksan 54596, Republic of Korea

**Keywords:** H9N2 avian influenza, Y280 lineage, recombinant HVT vaccine, maternally derived antibodies, viral replication, layer

## Abstract

The endemicity of H9N2 avian influenza viruses (AIVs), particularly the Y280 lineage, poses persistent challenges to the poultry industry due to the limitations of inactivated vaccines, such as interference by maternally derived antibodies (MDAs) and incomplete suppression of viral replication. This study evaluated the immunogenicity and protective efficacy of a novel recombinant turkey herpesvirus vaccine expressing the hemagglutinin gene of H9N2/Y280 (rHVT-H9/Y280) in commercial Hy-Line Brown layers with high-MDA backgrounds. In a comparative challenge study, the rHVT-H9/Y280 vaccine induced complete protection against a homologous Y280 strain challenge at 4 weeks of age, whereas commercial inactivated vaccines failed to completely block replication, showing virus isolation rates of 16.7–25%. Long-term serological monitoring demonstrated that the rHVT-H9/Y280 vaccine elicited a robust humoral response characterized by persistent maintenance of high HI titers (>8.0 log_2_) up to 39 weeks post-vaccination. These findings confirm that rHVT-H9/Y280 effectively overcomes MDA interference and provides protection by inhibition of viral replication in layer chickens, making it a promising candidate for the effective control of H9N2 AIV in endemic regions.

## Simple Summary

H9N2-subtype avian influenza (H9N2) is a widespread endemic disease causing significant economic losses in the global poultry industry. Currently, control relies mainly on inactivated vaccines, but their efficacy is often limited by interference from maternally derived antibodies (MDAs) in chicks and an inability to completely prevent virus spread. This study evaluated a new-generation vaccine, rHVT-H9/Y280, which uses a turkey herpesvirus vector to deliver H9N2 protection. We tested this vaccine in commercial layer chickens with high levels of MDA. The results showed that, unlike traditional vaccines, the rHVT-H9/Y280 vaccine was not affected by MDAs and provided 100% protection against the virus, completely blocking viral replication in internal organs. Furthermore, a single dose provided long-lasting immunity, with antibody levels persisting for up to 39 weeks. These findings suggest that this novel vaccine can effectively prevent infection and transmission even in young chicks with maternal immunity, helping to purify poultry flocks.

## 1. Introduction

H9N2-subtype low-pathogenicity avian influenza viruses (LPAIVs) have become deeply established in poultry populations across Eurasia and Africa since the mid-1990s, evolving into a widespread endemic disease that imposes a heavy economic burden on the global poultry industry [1]. Although characterized as low-pathogenicity, H9N2 infections are frequently associated with significant production losses, including severe egg drop syndromes and high mortality rates when exacerbated by secondary bacterial or viral coinfections [2,3]. Beyond their economic impact, H9N2 viruses pose a persistent zoonotic threat due to their ability to donate internal gene segments to other subtypes, thereby facilitating the genesis of novel reassortants with pandemic potential, such as the H5N1, H7N9, and H10N8 viruses that have caused human fatalities [4,5]. Furthermore, recent molecular surveillance indicates that many circulating H9N2 strains, particularly those of the Y280 lineage, have acquired mammalian-adaptive mutations (e.g., Q226L in the hemagglutinin receptor-binding site), which enhance their binding affinity to human-type alpha2,6-linked sialic acid receptors [6,7].

Currently, the primary control strategy for H9N2 relies on the administration of oil-emulsion inactivated whole-virus vaccines [8,9]. While these vaccines can mitigate clinical signs, they exhibit critical limitations that hinder the eradication of the disease [8,9]. First, inactivated vaccines primarily induce humoral immunity (IgG) and are often unable to elicit sufficient mucosal immunity (IgA) or cell-mediated immunity (CMI) to prevent viral shedding, allowing “silent replication” and continued transmission within vaccinated flocks [8,9,10]. Second, the efficacy of inactivated vaccines is severely compromised by maternally derived antibodies (MDAs) in young chicks, which neutralize the vaccine antigen before active immunity can be established [11,12]. Third, the inability to distinguish between infected and vaccinated animals (DIVA) using conventional serological methods complicates surveillance and eradication efforts [11,12].

To address these challenges, recombinant turkey herpesvirus (rHVT) has emerged as a promising vector platform for next-generation avian influenza vaccines [13,14]. As a cell-associated virus, HVT can evade neutralization by MDAs, enabling effective immunization in day-old chicks regardless of maternal antibody levels [13,15]. Furthermore, rHVT-based vaccines expressing the hemagglutinin (HA) gene have been shown to induce robust cellular and humoral immune responses, providing long-lasting protection and significantly reducing viral replication compared to inactivated vaccines [13,14,16]. Additionally, rHVT vaccines inherently support a DIVA strategy, as vaccinated birds seroconvert only against the inserted HA protein and remain negative for other influenza viral proteins [13,17].

In light of the recent invasion of the antigenically distinct Y280 lineage H9N2 virus across Asian countries and the inadequacies of existing control measures, there is an urgent need for an updated, highly efficacious vaccine. This study aims to evaluate the protective efficacy of a novel rHVT-H9/Y280 vaccine in commercial layers possessing high levels of maternal antibodies [18,19]. Specifically, we assessed the vaccine’s ability to induce protective immunity, block viral replication, and offer superior protection compared to commercial inactivated vaccines, thereby validating its potential as a strategic tool for the control and eventual eradication of the emerging H9N2 Y280 lineage.

## 2. Materials and Methods

### 2.1. Experimental Chickens

Commercial Hy-Line Brown layer chickens were used in two independent studies. These birds were sourced from a breeder flock that had been routinely immunized with inactivated H9N2 vaccines. All birds were housed in biosecurity level 2 (BSL-2) isolation units with controlled environmental conditions. Water and feed were provided ad libitum throughout the experimental periods. All experimental and animal management procedures were undertaken in accordance with the requirements of the Animal Care and Ethics Committee of Jeonbuk National University and the animal facility at Jeonbuk National University is fully accredited by the National Association of Laboratory Animal Care (approval number: NON2023-008).

### 2.2. Vaccines

The investigational vaccine, rHVT-H9/Y280, is a cell-associated recombinant turkey herpesvirus (HVT) vector vaccine expressing the hemagglutinin (HA) gene of the Y280-lineage H9N2 virus (strain A21-MRA-003) [20]. For comparison, three commercially available inactivated oil-emulsion vaccines were utilized: Commercial vaccine 1 (containing H9N2/Y280 strain), Commercial vaccine 2 (containing H9N2/Y280 strain), and Commercial vaccine 3 (containing H9N2/Y280 and IBV strains).

### 2.3. Study I: Protective Efficacy Against Avian Influenza Virus Challenge

Experimental design: Sixty-six 14-day-old chickens were randomly distributed into six groups. Group 1 (*n* = 12) was immunized via the subcutaneous (s.c.) route with 1000 PFU/0.2 mL of the rHVT-H9/Y280 vaccine. Groups 2, 3, and 4 (*n* = 12 per group) received a single dose (0.5 mL) of Commercial Vaccines 1, 2, and 3, respectively, via the intramuscular (i.m.) route. Group 5 (*n* = 10, Positive Control) and Group 6 (*n* = 8, Negative Control) were mock-vaccinated with diluent injections of PBS (200 μL) in the neck.

Challenge and sampling: At 4 weeks post-vaccination (4 WPV, 6 weeks of age), birds in Groups 1–5 were challenged intranasally with 10^7.0^ EID_50_ per bird of the A21-MRA-003 virus, while Group 6 remained unchallenged. Clinical signs and mortality were monitored daily for 5 days. To assess protection against viral replication, necropsies were performed at 5 days post-challenge (5 dpc), and cecal tonsils (CT) were collected to quantify the viral load. H9N2 virus isolation in cecal tonsils was quantified by titrating homogenized tissue samples in 10-day-old specific pathogen-free (SPF) embryonated chicken eggs. Viral titers were calculated using the Reed–Muench method and expressed as log_10_ EID_50_/mL. The Protection Index (PI) was calculated based on the reduction in the proportion of virus-positive birds compared to the positive control group [20]. PI = 100% × [(positive isolation rate in Control − positive isolation rate in Vaccinated)/positive isolation rate in Control].

### 2.4. Study II: Duration of Immunity and Vector Kinetics

Experimental design: twenty one-day-old chicks were assigned to two groups (*n* = 10 per group). Group 1: Immunized s.c. with 1000 PFU/0.2 mL of rHVT-H9/Y280. Group 2: Immunized i.m. with 0.5 mL of the Commercial H9N2/Y280-IBV vaccine. Blood samples were collected weekly from 1 to 5 WPV, and subsequently at weeks 8, 10, 12, 14, 16, 18, 20, and periodically up to 39 WPV to monitor the persistence of humoral immunity. Serum antibody titers were quantified using the hemagglutination inhibition (HI) assay according to standard protocols [20,21]. Briefly, chicken sera were two-fold serially diluted in duplicate in 96-well V-bottom plates and incubated with 4 hemagglutination units (HAU) of H9N2/Y280 antigen diluted in PBS at room temperature for 30 min. Subsequently, 0.5% chicken red blood cells were added and incubated for an additional 30 min. The HI antibody titer was defined as the reciprocal of the highest serum dilution that completely inhibited hemagglutination.

To verify the systemic persistence of the HVT vector, peripheral blood mononuclear cells (PBMCs) were isolated from blood samples collected at 3 and 20 WPV [22]. The replication and persistence of the rHVT vector in PBMCs were quantified using a specific real-time PCR (qPCR) assay targeting the HVT genome [22]. Results were analyzed to confirm the establishment of viremia, which is critical for the induction of long-term immunity.

### 2.5. Statistical Analysis

Data were analyzed using one-way analysis of variance (ANOVA) followed by Tukey’s multiple comparison test for differences between groups, using SPSS software (version 19.0; IBM Corp., Armonk, NY, USA). A *p*-value of <0.05 was considered statistically significant.

## 3. Results

### 3.1. Humoral Immune Response to Vaccination

**Figure 1:**
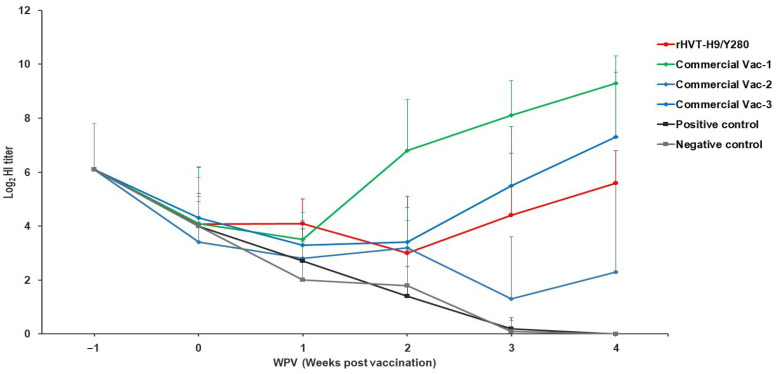
Kinetics of hemagglutination inhibition (HI) antibody titers in commercial layers. The *X*-axis represents weeks post-vaccination (WPV), and the *Y*-axis represents the mean HI antibody titer (log_2_). Data are expressed as mean ± standard deviation (SD). Group assignments: G1 (red line) = rHVT-H9/Y280; G2–G4 (blue, green, and gray lines) = Commercial Inactivated Vaccines 1, 2, and 3; G5–G6 = positive and negative controls (PBS).

At the initial stage of the experiment (14 days of age, 0 WPV), all experimental groups (G1–G6) exhibited a background of maternally derived antibodies (MDA), with mean hemagglutination inhibition (HI) titers ranging from 3.4 ± 1.5 to 4.3 ± 0.8 log_2_ (Figure 1). Longitudinal monitoring of the non-immunized control groups (G5, G6) determined the half-life of H9N2 MDA in this study to be approximately 5.1 days. By 3 weeks post-vaccination (3 WPV, 35 days of age), antibody titers in the control groups had declined to baseline levels (approaching 0), indicating that MDA was fully catabolized prior to challenge. Against this background, the recombinant vaccine group (G1, rHVT-H9/Y280) demonstrated robust immune response kinetics following a single subcutaneous (S.C.) administration. Despite slight interference from residual MDA during the early post-vaccination phase (2 WPV), antibody levels in this group showed a sustained increasing trend. By 4 weeks post-vaccination (4 WPV, the day of challenge), the mean HI titer reached 5.6 ± 1.2 log_2_, with a seroconversion rate of 100% (12/12), confirming that rHVT-H9/Y280 can induce effective humoral immunity even in the presence of MDA interference. In contrast, the commercial inactivated vaccine groups (G2–G4) displayed significant heterogeneity in immunogenicity: the G2 group (Commercial H9N2/Y280) induced the highest humoral response (9.3 ± 1.0 log_2_ at 4 WPV), and the G4 group performed well (7.3 ± 2.4 log_2_); however, the G3 group showed suboptimal efficacy with a mean titer of only 2.3 ± 3.3 log_2_ and a low seroconversion rate of 33.3%.

### 3.2. Efficacy Against H9N2 Challenge

To evaluate the clinical protective efficacy of the vaccines, all experimental chickens were challenged intranasally at 42 days of age (4 WPV). Viral isolation was assessed in cecal tonsils (CT) collected at 5 days post-challenge (5 DPC). The results demonstrated that the rHVT-H9/Y280 group (G1) completely inhibited the replication of the challenge virus in target organs; no virus was detected in the cecal tonsils of any tested birds (12/12) (Table 1). In contrast, although some commercial inactivated vaccine groups (e.g., G2) possessed extremely high circulating antibody titers prior to challenge, they failed to provide complete protection against virus replication. Viral replication was detected in the cecal tonsils of groups G2, G3, and G4, with virus isolation rates of 16.7%, 16.7%, and 25%, respectively, and mean viral loads ranging from 2.0 ± 2.2 log_10_ EID_50_/mL. The Protection Index (PI) was calculated based on virus isolation results: rHVT-H9/Y280 (G1) achieved a protection level of 100% and the commercial vaccine groups achieved lower levels (G2: 72.2%, G3: 72.2%, G4: 58.3%).

### 3.3. Long-Lasting Humoral Immune Response and Virus Kinetics in Layers

**Figure 2:**
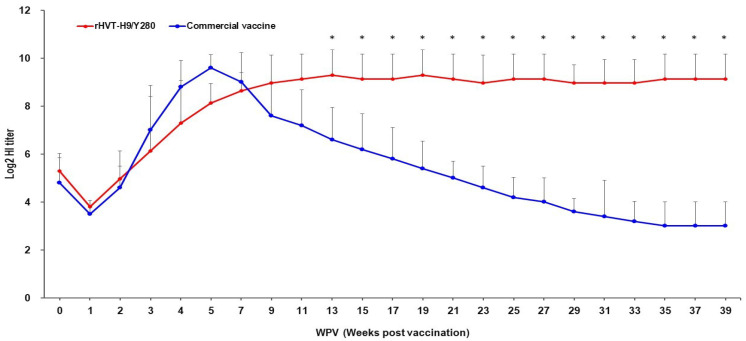
Long-term persistence of humoral immunity in commercial layers. The *X*-axis represents weeks post-vaccination (WPV), and the *Y*-axis represents the mean HI antibody titer (log_2_). Data are expressed as mean ± standard deviation (SD). Group assignments: rHVT-H9/Y280 (red line) and Commercial Inactivated Vaccine (blue line). Significant differences (*p* < 0.05) between the two groups are indicated by asterisks (*) at the corresponding time points.

In order to evaluate the potential of the rHVT-H9/Y280 vector in layers, we inoculated day-old chicks with the rHVT-H9/Y280 candidate or a commercial inactivated vaccine (Y280/IB). Their serum antibody responses to the H9 antigen were assessed by HI tests for 39 weeks. Average titers of the HI antibody of each vaccine group are shown in Figure 2. The HI antibody titer of the rHVT-H9/Y280 group showed a kinetic profile of steady rise and persistent maintenance throughout the experiment. The titer of antibody to H9 continuously increased up to 11 WPV, reaching > 8.0 log_2_, and then this high antibody titer persisted in layers until the end of the experiment (39 WPV). In contrast, the antibody kinetic curve of the commercial inactivated vaccine group showed a peak antibody titer (9.8 log_2_) at 5 WPV, and then the titer gradually decreased to approximately 3.0 log_2_ by the end of the experiment. Quantification of HVT viral load in PBMCs at 3 WPV and 20 WPV confirmed persistent infection, with mean viral copy numbers consistently maintained between 10^3.3^ and 10^3.5^ per 10^6^ PBMCs.

## 4. Discussion

Maternally derived antibodies (MDAs) represent a double-edged sword in poultry immunology: while they provide essential early protection for chicks, they constitute a primary barrier to immunization failure against H9N2 avian influenza [8,12]. Multiple studies have demonstrated that high levels of MDAs significantly interfere with the humoral immune response to inactivated vaccines [23,24,25,26,27]. Consistent with these observations, our data confirm that viral replication persists even in the presence of high vaccine-induced HI titers, highlighting the limitations of relying solely on humoral immunity for H9N2 control [28].

The superior efficacy of rHVT-H9/Y280 is largely attributed to its unique biological mechanisms. As a cell-associated virus, the spread of HVT within the host is highly dependent on cell-to-cell contact, with viral particles rarely exposed to extracellular fluids [13]. This mode of transmission effectively evades neutralization by high concentrations of maternal IgG in the blood, allowing the vaccine virus to successfully colonize and establish infection even in chicks with high MDA titers [18,29]. Belonging to the herpesvirus family, HVT establishes lifelong latency in avian T lymphocytes. During latency, the viral genome not only persists but also undergoes intermittent reactivation, expressing the inserted foreign gene (H9 HA) [13]. This mechanism functions effectively as a micro-reservoir continuously releasing antigens to stimulate the immune system. This observation perfectly explains the antibody kinetics observed in Figure 2: unlike inactivated vaccines where titers decay rapidly, antibody levels in the rHVT group rose steadily with age and were maintained at high levels long-term (>8.0 log_2_ at 39 weeks). Distinct from inactivated vaccines which primarily activate the MHC-II pathway to produce antibodies, rHVT, as a live viral vector, expresses HA proteins synthesized intracellularly. These can be processed and presented via the MHC-I pathway, thereby potently activating CD8+ cytotoxic T lymphocyte (CTL) responses [30,31,32]. This cell-mediated immunity is crucial for clearing intracellular viruses and is the key factor enabling the complete clearance of virus from the cecal tonsils (intestinal mucosal tissue) observed in this study. However, it should be noted that the present study did not directly quantify specific markers of cell-mediated immunity. Future studies will be designed to systematically evaluate these cellular immune responses in order to further elucidate the mechanisms underlying the superior efficacy of the rHVT-H9/Y280 vaccine.

From an epidemiological perspective, the ability of rHVT-H9/Y280 to block viral replication is critical, making it a reliable tool for H9N2 control. By severing the horizontal transmission chain, this vaccine minimizes environmental contamination and reduces the evolutionary pressure for antigenic drift [2,12]. Additionally, given that the Y280 lineage of H9N2 has acquired the ability to bind human receptors (Q226L mutation), the risk of cross-species transmission cannot be ignored [2,7]. By blocking viral replication at the source (poultry), this vaccine provides a solid barrier against zoonotic risks.

While this study confirms the excellent efficacy of rHVT-H9/Y280 in layers, several critical aspects warrant further investigation in future studies. Firstly, viral replication was evaluated solely by virus isolation from cecal tonsils, a major site of avian influenza virus persistence. However, oropharyngeal and cloacal swabs were not collected. Therefore, future studies incorporating multiple sampling sites will be necessary to definitively determine whether viral replication and transmission are completely inhibited. Secondly, given that the long growth cycle of layers favors the gradual establishment of HVT-induced immunity, further data is required to determine whether this vaccine can provide sufficient protection within the short production window of broiler chickens [18,29]. Thirdly, regarding the sample size, the inherent individual variability in maternal antibody levels within commercial flocks suggests that future large-scale field trials would be beneficial to further validate the statistical robustness of these findings. Fourth, with the widespread application of various recombinant HVT vaccines (e.g., rHVT-ND, rHVT-ILT), the potential for vector interference during concurrent administration—which could compromise antigen expression efficiency—requires the evaluation of optimized combined immunization strategies [30]. Finally, despite the robust immune platform provided by HVT, the inserted HA gene must remain antigenically matched to circulating strains; thus, utilizing technologies such as CRISPR/Cas9 to rapidly update vaccine strains in response to the rapid evolution of the G1, Y280 and Y439 lineage will likely become a standard component of future vaccine development [12,30,31].

## 5. Conclusions

In summary, this study demonstrates that the novel rHVT-H9/Y280 vector vaccine exhibits protective efficacy superior to traditional inactivated vaccines in commercial layers with high-maternal-antibody backgrounds. First, utilizing the cell-associated characteristics of the HVT vector, the vaccine successfully bypassed maternal antibody neutralization, establishing a solid immune foundation in all immunized birds. Second, through the synergy of humoral and cellular immunity, the vaccine completely blocked viral replication in the cecal tonsils, thereby addressing the transmission-blocking shortcomings of inactivated vaccines. Third, the vaccine induced a durable immune response lasting up to 39 weeks with steadily rising antibody levels, avoiding the rapid antibody decay associated with inactivated vaccines and greatly simplifying the immunization schedule. Fourth, as a genetically engineered vaccine, it naturally possesses DIVA characteristics [30], which aids future disease control programs. Therefore, the rHVT-H9/Y280 vaccine provides a powerful and reliable novel tool for severing the transmission chain and controlling the H9N2 Y280 lineage in endemic regions.

## Figures and Tables

**Table 1:** Protective efficacy of the used vaccines against H9N2/Y280 challenge.

Group	Virus Isolation ^a^ (%)	Virus Load (log_10_ EID_50_/mL)	Protection Index (%)
G1 (rHVT-H9/Y280)	0% (0/12)	Not Detected	100
G2 (Commercial Vac 1)	16.7% (2/12)	2.2 ± 0.5	72.2
G3 (Commercial Vac 2)	16.7% (2/12)	2.0 ± 0.6	72.2
G4 (Commercial Vac 3)	25% (3/12)	2.2 ± 0.8	58.3
G5 (Pos Ctrl)	60% (6/10)	2.0 ± 0.5	-
G6 (Neg Ctrl)	0% (0/8)	Not Detected	-

^a^ Virus isolation was performed by inoculating CT sample in SPF embryonated chicken eggs.

## Data Availability

The original contributions presented in this study are included in the article. Further inquiries can be directed to the corresponding authors.
